# Supplementary material for: Time-series transcriptome analysis identified differentially expressed genes in broiler chicken infected with mixed Eimeria species
Source: Front Genet. 2022 Aug 8;13:886781. doi: 10.3389/fgene.2022.886781 (PMC9393255; doi:10.3389/fgene.2022.886781)
Supplement: Supplementary file 2 [file DataSheet1.ZIP › 7dpi_GO.Gsea.1625106495923/GOMF_PROTEIN_PHOSPHORYLATED_AMINO_ACID_BINDING.html]

Details for gene set GOMF\_PROTEIN\_PHOSPHORYLATED\_AMINO\_ACID\_BINDING[GSEA]

|  || Dataset | TMM\_7dpi\_gct\_format\_7dpi\_gct\_format.Class\_4dpi.cls #PC\_versus\_NC.Class\_4dpi.cls #PC\_versus\_NC\_repos |
| Phenotype | Class\_4dpi.cls#PC\_versus\_NC\_repos |
| Upregulated in class | 0 |
| GeneSet | GOMF\_PROTEIN\_PHOSPHORYLATED\_AMINO\_ACID\_BINDING |
| Enrichment Score (ES) | -0.56257933 |
| Normalized Enrichment Score (NES) | -1.8289934 |
| Nominal p-value | 0.0 |
| FDR q-value | 0.109830104 |
| FWER p-Value | 0.8694 |
Table: GSEA Results Summary

  

Fig 1: Enrichment plot: GOMF\_PROTEIN\_PHOSPHORYLATED\_AMINO\_ACID\_BINDING      
 Profile of the Running ES Score & Positions of GeneSet Members on the Rank Ordered List

  

| SYMBOL | TITLE | RANK IN GENE LIST | RANK METRIC SCORE | RUNNING ES | CORE ENRICHMENT || 1 | BCAR3 | na | 1387 | 0.408 | -0.1010 | No |
| 2 | PIN1 | na | 2687 | 0.165 | -0.2035 | No |
| 3 | SHE | na | 3573 | 0.055 | -0.2755 | No |
| 4 | HCK | na | 4481 | -0.048 | -0.3495 | No |
| 5 | YWHAB | na | 4566 | -0.057 | -0.3545 | No |
| 6 | YWHAE | na | 4774 | -0.078 | -0.3689 | No |
| 7 | PCIF1 | na | 5244 | -0.125 | -0.4035 | No |
| 8 | SHC1 | na | 5796 | -0.179 | -0.4430 | No |
| 9 | FBXW7 | na | 6468 | -0.250 | -0.4900 | No |
| 10 | ABL1 | na | 6549 | -0.259 | -0.4872 | No |
| 11 | GRB2 | na | 6686 | -0.274 | -0.4886 | No |
| 12 | CRK | na | 6900 | -0.297 | -0.4955 | No |
| 13 | SCAF4 | na | 7002 | -0.308 | -0.4927 | No |
| 14 | CRKL | na | 7144 | -0.324 | -0.4927 | No |
| 15 | LEO1 | na | 7676 | -0.381 | -0.5231 | No |
| 16 | RTF1 | na | 8038 | -0.427 | -0.5377 | No |
| 17 | SAMSN1 | na | 8162 | -0.441 | -0.5319 | No |
| 18 | LDLRAP1 | na | 8296 | -0.457 | -0.5263 | No |
| 19 | STAP1 | na | 8317 | -0.461 | -0.5111 | No |
| 20 | SCAF8 | na | 8928 | -0.539 | -0.5424 | Yes |
| 21 | SHB | na | 9035 | -0.554 | -0.5311 | Yes |
| 22 | FGR | na | 9413 | -0.617 | -0.5401 | Yes |
| 23 | BTRC | na | 9430 | -0.620 | -0.5188 | Yes |
| 24 | PIK3R2 | na | 9597 | -0.645 | -0.5091 | Yes |
| 25 | ZAP70 | na | 9604 | -0.646 | -0.4860 | Yes |
| 26 | SYK | na | 9655 | -0.655 | -0.4663 | Yes |
| 27 | NEDD4 | na | 9675 | -0.657 | -0.4439 | Yes |
| 28 | YES1 | na | 9689 | -0.659 | -0.4209 | Yes |
| 29 | PTPN6 | na | 9763 | -0.668 | -0.4026 | Yes |
| 30 | SH2D3C | na | 9985 | -0.709 | -0.3952 | Yes |
| 31 | MAPK3 | na | 10320 | -0.774 | -0.3948 | Yes |
| 32 | PTPN11 | na | 10560 | -0.822 | -0.3848 | Yes |
| 33 | PTPN3 | na | 10579 | -0.828 | -0.3561 | Yes |
| 34 | LCK | na | 10662 | -0.845 | -0.3321 | Yes |
| 35 | PLCG2 | na | 10697 | -0.853 | -0.3037 | Yes |
| 36 | SH3BP2 | na | 10828 | -0.887 | -0.2822 | Yes |
| 37 | CBLB | na | 10996 | -0.935 | -0.2620 | Yes |
| 38 | PIK3R3 | na | 11045 | -0.949 | -0.2314 | Yes |
| 39 | RASA1 | na | 11049 | -0.950 | -0.1970 | Yes |
| 40 | SOCS3 | na | 11181 | -0.994 | -0.1716 | Yes |
| 41 | PIK3R1 | na | 11265 | -1.023 | -0.1412 | Yes |
| 42 | ABL2 | na | 11336 | -1.051 | -0.1087 | Yes |
| 43 | SHF | na | 11658 | -1.206 | -0.0915 | Yes |
| 44 | CBL | na | 11873 | -1.446 | -0.0566 | Yes |
| 45 | VAV2 | na | 11979 | -1.860 | 0.0026 | Yes |
Table: GSEA details [plain text format]

  

Fig 2: GOMF\_PROTEIN\_PHOSPHORYLATED\_AMINO\_ACID\_BINDING      
 Blue-Pink O' Gram in the Space of the Analyzed GeneSet

  

Fig 3: GOMF\_PROTEIN\_PHOSPHORYLATED\_AMINO\_ACID\_BINDING: Random ES distribution      
 Gene set null distribution of ES for **GOMF\_PROTEIN\_PHOSPHORYLATED\_AMINO\_ACID\_BINDING**
